# Microduplication in the 2p16.1p15 chromosomal region linked to developmental delay and intellectual disability

**DOI:** 10.1186/s13039-018-0388-y

**Published:** 2018-06-20

**Authors:** Luca Lovrecic, Chiara Gnan, Federica Baldan, Alessandra Franzoni, Sara Bertok, Giuseppe Damante, Bertrand Isidor, Borut Peterlin

**Affiliations:** 1 Clinical Institute of Medical Genetics, University Medical Center Ljubljana, Ljubljana, Slovenia; 2 Istituto di Genetica Medica, Azienda Ospedaliero-Universitaria di Udine, Udine, Italy; 3 Dipartimento di Medicina Interna e Specialità Mediche dell’Università Sapienza di Roma, Udine, Italy; 4 Department of Pediatric Endocrinology, Diabetes and Metabolic Diseases, University Children’s Hospital, University Medical Center Ljubljana, Ljubljana, Slovenia; 5 Dipartimento di Area Medica, Università di Udine, Udine, Italy; 6 Service de génétique médicale, CHU de Nantes, Nantes, France

**Keywords:** 2p16.1p15, Molecular karyotyping, Array CGH, microduplication, Duplication, Developmental delay, Macrocephaly

## Abstract

**Background:**

Several patients with the 2p16.1p15 microdeletion syndrome have been reported. However, microduplication in the 2p16.1p15 chromosomal region has only been reported in one case, and milder clinical features were present compared to those attributed to 2p16.1p15 microdeletion syndrome. Some additional cases were deposited in DECIPHER database.

**Case presentation:**

In this report we describe four further cases of 2p16.1p15 microduplication in four unrelated probands. They presented with mild gross motor delay, delayed speech and language development, and mild dysmorphic features. In addition, two probands have macrocephaly and one a congenital heart anomaly. Newly described cases share several phenotype characteristics with those detailed in one previously reported microduplication case.

**Conclusion:**

The common features among patients are developmental delay, speech delay, mild to moderate intellectual disability and unspecific dysmorphic features. Two patients have bilateral clinodactyly of the 5th finger and two have bilateral 2nd-3rd toes syndactyly. Interestingly, as opposed to the deletion phenotype with some cases of microcephaly, 2 patients are reported with macrocephaly. The reported cases suggest that microduplication in 2p16.1p15 chromosomal region might be causally linked to developmental delay, speech delay, and mild intellectual disability.

## Background

The implementation of molecular karyotyping (array CGH; aCGH) into the routine genetic diagnostics of children with intellectual disability/developmental delay with or without various congenital anomalies and dysmorphic features has considerably increased the diagnostic utility of genetic testing, as well as the pace of identification of novel microdeletion and microduplication syndromes [1, 2]. In parallel, it enabled us to further delineate genes or groups of genes whose deletion or duplication is associated with a specific phenotype; the recurrent microdeletion and microduplication syndromes.

Herein reported region of 2p16.1p15 was identified as a new microdeletion syndrome (OMIM #612513) already in 2007, when two patients with 4.5 Mb and 5.7 Mb de novo deletions were reported [3]. Since then more than 10 additional patients with different deletion sizes and breakpoints were reported in the literature, with the clinical phenotype including different levels of intellectual disability and developmental delay, speech delay, microcephaly, structural brain anomalies, neuromotor deficits, visual impairment, strabismus, renal anomalies, camptodactyly, and dysmorphic features [4–10].

On the other hand, the microduplication of the same region 2p16.1p15 has only been recently described in one case [11]. The authors report a relatively milder clinical phenotype as compared to the above mentioned clinical features of the individuals with the deletion in this chromosomal region. As only one case has been extensively described, the phenotype associated to the 2p16.1p15 microduplication has not been delineated as a specific disorder.

**We present four further cases of microduplication 2p16.1p15 and** compare the clinical features of our patients with those previously described. In addition, some patients are described in DECIPHER and ClinGen databases and are included in the report as well (Fig. 2/Table 1). With growing number of patients further evidence is provided that 2p16.1p15 microduplication might be linked to developmental delay, speech delay, and mild intellectual disability.

**Table 1:** Clinical characteristics of previously reported cases with overlapping duplications

Case ID	Our case 1	Our case 2	DECIPHER 323264	DECIPHER 258333	Mimouchi-Bloch A, et al. (ref)	DECIPHER 1570	DECIPHER 265052
Coordinates of duplications (hg19)	Chr2:60113626_62111114	Chr2:60308869_62368583	Chr2:60236241–61,848,845	Chr2:59938734–62,025,519	Chr2:60150427_61816209	Chr2:60648296–61,568,645	Chr2:60541781–61,952,880
Chromosome band	2p16.1p15	2p16.1p15	2p16.1p15	2p16.1p15	2p16.1p15	2p16.1p15	2p16.1p15
Gender*	M, 3y	M, 5y	F, 15y	M	M, 5y	F, 3y	F, 7y
Developmental delay	+**	+	+	+	+	+	+
Speech delay	+	+	+	+	+	+	+
Intellectual disability	+	+	+	+	mild	+	-
Autism	-	-	-	-	- (ADHD)	-	-
Craniofaci al signs		Macrocephaly		Macrocephaly			
Forehead	Receding forehead		-			Frontal bossing	-
Eyes	Epicanthal folds Straight eyelashes, sparse eyebrows	Upslanted palpebral fissures	-		Puffy eyelids	Blepharophimosis Upslanted palpebral fissures	- -
Nose	Broad and high nasal bridge	Concave nasal bridge	-			Wide nasal bridge	-
Ears			-		Right earlobe		
Mouth	Pronounced philtrum Pronounced Cupid’s bow	Pronounced philtrum Pronounced Cupid’s bow	-	Small ears	sinus Broad philturm	Low-set ears Thin upper	
						and lower lip vermilion Micrognathia	
Other Cardiovascular	–	Atrial septum defect	–	–	–	Atrial septum defect	–
Hypotonia	+	+	-	+	-	-	-
Other	Clinodactyly of the 5th finger, bilaterally 2nd-3rd toes syndactyly	Clinodactyly of the 5th finger	Epilepsy Obesity	Recurrent infections Abnormality of erythrocytes Small hands	Visual impairment	Short stature Short palm Tapered fingers 2nd-3rd toe syndactyly	Obesity Arthritis Precocious puberty

^*^ *M* male, *F* female, ** + present, − not reported

## Cases presentation

### Patient 1

The first patient is a male child of healthy non consanguineous parents. He was born at term after an uneventful pregnancy with birth weight 3.244 g (25-50P), birth length 50.3 cm (25-50P) and head circumference 38.5 cm (97P). At birth he presented mild jaundice, reduced movements and some initial feeding difficulties. Thoracic and abdominal ultrasound were normal.

**Fig. 1:**
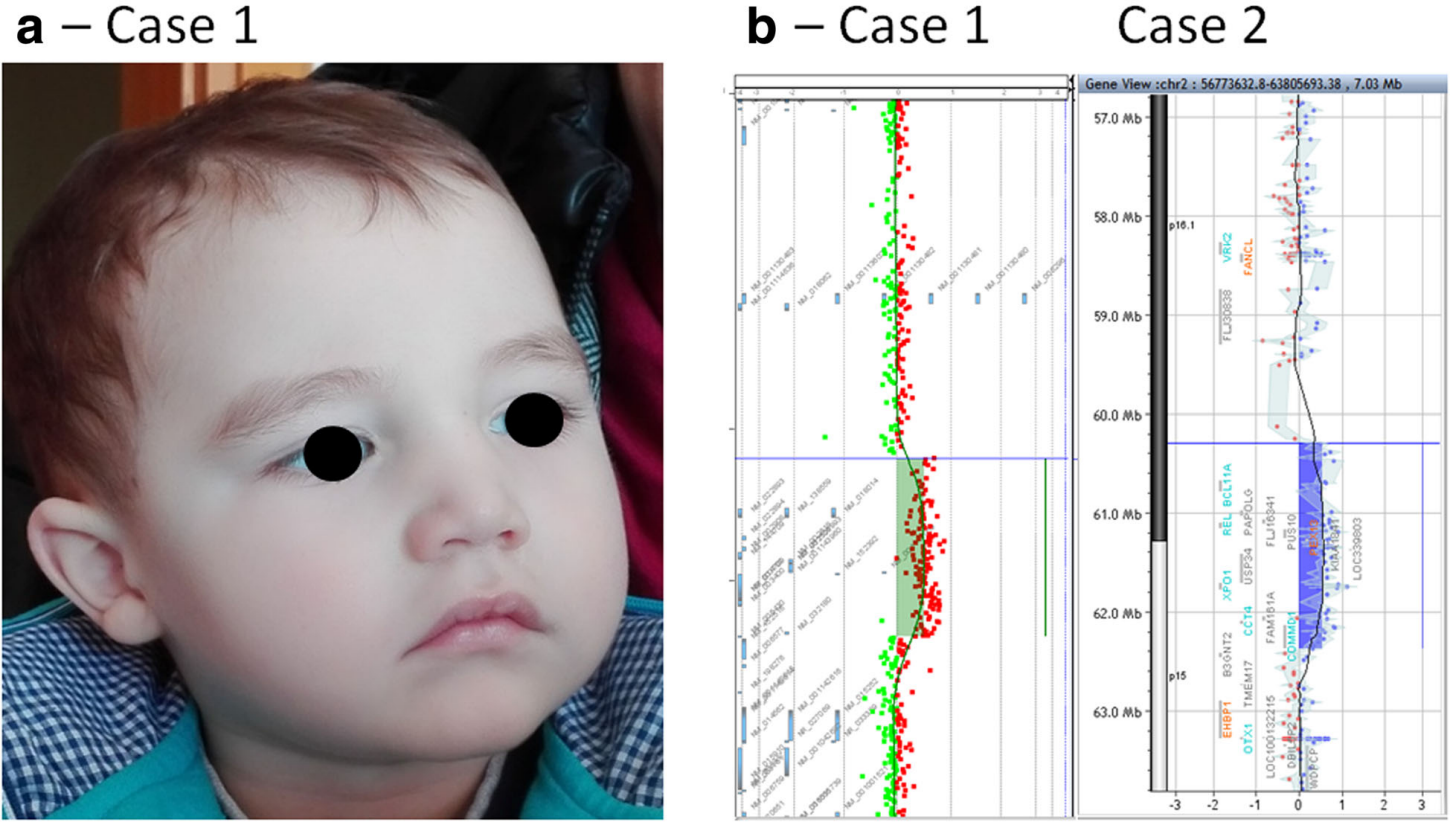
Facial dysmorphism of case 1 and the array CGH profiles in both probands. **a** The details about facial dysmorphis are presented in Table 1; **b** The array CGH results are presented for both Case 1 and Case 2. The reported duplications are almost the same. Case 1 - arr[GRCh37] 2p16.1p15(60113626_62111114)× 3 dn; Case 2 - arr[GRCh37] 2p16.1p15(60308869_62368583)× 3 dn

The developmental milestones in the boy were delayed - he was able to sit independently at 11 months of age and he started to walk at 21 months. The first sounds and signs of speech/language development were described after 14 months of age. At 4 years of age he was not able to generate complete sentences. A mild intellectual disability was present. His growth parameters were within the normal range. Mild dysmorphic features were described - receding forehead, broad and high nasal bridge, sparse eyebrows, epicanthal folds, straight eyelashes and pronounced philtrum (Fig. 1a). He has bilateral clinodactyly of the 5th finger and bilateral 2nd-3rd toes syndactyly. He was referred to genetic counseling for delayed global development. Informed consent for the study was obtained from the parents.

Microarray analysis (180 K CGH array, Agilent Technologies-Fig. 1b) revealed a de novo microduplication of 2,00 Mb in chromosome 2p16.1p15 region (arr[GRCh37] 2p16.1p15(60113626_62111114)× 3 dn). No other pathogenic genomic imbalance was detected in the proband’s sample.

### Patient 2

The second patient is a 5-year old boy, born as a first child to healthy non-consanguineous parents. The mother reported two previous early spontaneous abortions. Otherwise, the family history is unremarkable. He was born after an uneventful pregnancy in the 37th week of gestation after a spontaneous start of the delivery. The boy’s birth weight was 2430 g (10-25P), birth length 46 cm (10-25P), and head circumference 34.5 cm (75-90P). He had gastroesophageal reflux in the first few months, the abdominal ultrasound was normal. Due to apnoic attacks the boy was administered to hospital at the age of 5 months. The pH-metry confirmed gastroesophageal reflux, ECG and CMCRF were normal. The neurologist described a mild hypertonus and related mild motor delay. He sat independently at 9 months of age and he started walking at 20 months of age. The parents noted shortness of breath and tiredness after simple physical tasks, therefore, he was evaluated by a paediatric cardiologist. Two haemodynamically significant ASDs were noted and a slightly dilated right ventricle; corrective surgery is planned. The tests of acylcarnitine profiles and aminoacids in blood and organic acid in urine were normal. At the age of four his height and weight were in the normal range (height 99.2 cm (17P), weight 16.1 kg (46P)), however, the head circumference showed macrocephaly - 53.8 cm (>97P).

Microarray analysis (180 K CGH array, Agilent Technologies- Fig. 1b) revealed a de novo microduplication of 2.06 Mb in chromosome 2p16.1p15 region (arr[GRCh37] 2p16.1p15(60308869_62368583)× 3 dn). No other pathogenic genomic imbalance was detected in the proband’s sample.

### Patient 3 - DECIPHER ID323264

This male patient was born after an uneventful pregnancy in the 36th week of gestation with birth weight 2820 g (50P), birth length 51 cm (90P), and head circumference 36.5 cm (97P). He walked at 18 months and showed speech delay. He has learning difficulties and attends a special education program. At 15 years his growth parameters were in the normal range (weight 60 kg (50-75P), height 165 cm (25-50P), head circumference 55 cm (25P). Microarray analysis revealed a microduplication of 1.61 Mb in chromosome 2p16.1p15 region, confirmed by FISH (arr[GRCh37] 2p16.1p15(60236241–61,848,845)× 3 mat). Also, his mother was a carrier of the duplication with learning difficulties, epilepsy and obesity. No further details about her phenotype were available.

### Patient 4 - DECIPHER ID258333

This male patient was born at term after an uneventful pregnancy. He displayed early hypotonia and neonatal feeding difficulties. He walked at 20 months and showed speech delay. Later, moderate intellectual disability was diagnosed. In the childhood, he had recurrent infections with neutropenia and thrombocytopenia. He has small ears and small hands. At 33 years his growth parameters were as follows: height 192 cm (95P), weight 95 kg (95P), head circumference 63.5 cm (>97P). Microarray analysis revealed a microduplication of 2.09 Mb in chromosome 2p16.1p15 region, confirmed by FISH (arr[GRCh37] 2p16.1p15(59938734–62,025,519)× 3 dn).

## Comparison with overlapping cases

The Database of Genomic Variants (DGV) [12] was checked for the presence of similar CNVs in control populations and none were revealed. A search through several databases, DECIPHER [13], ClinGen [14] and scientific literature indexed in PubMed (http://www.ncbi.nlm.nih.gov/pubmed) was performed.

**Fig. 2:**
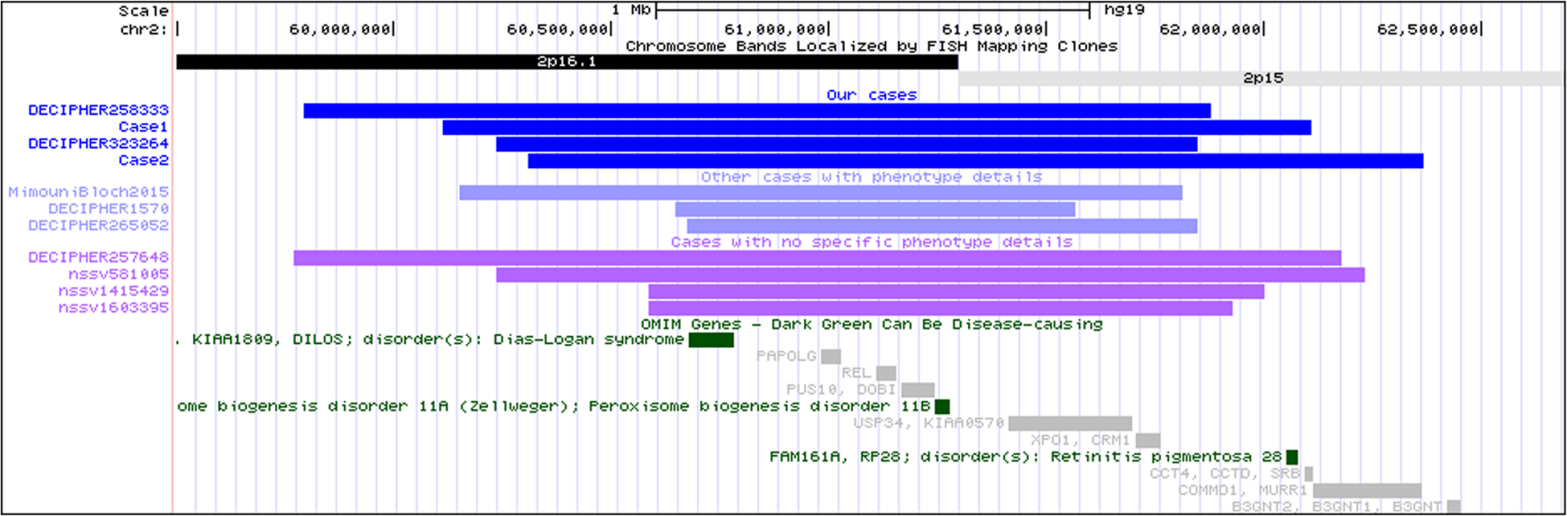
The duplication overlap with other reported/deposited casesSizes and locations of herein reported cases and other reported overlapping cases are shown. The UCSC Genome Browser is used. Gene content (OMIM genes) is presented under the reported cases

According to all cases reported in the literature or deposited in databases with phenotype description (Table 1), there are 3 additional cases with overlapping duplications. One presented in detail as a case report [11] and 2 deposited in DECIPHER database. All were de novo. There are 4 additional cases, 1 in DECIPHER database with no phenotype data and 3 in ClinGen/ISCA database, listed under category “Developmental delay and additional significant developmental and morphological phenotypes referred for genetic testing”. All CNVs are presented in Fig. 2.

The common features among the 7 patients with described phenotypic details are developmental delay, speech delay, mild to moderate intellectual disability, and unspecific dysmorphic features. Two patients have bilateral clinodactyly of the 5th finger and two have bilateral 2nd-3rd toes syndactyly. Interestingly, as opposed to the deletion phenotype**,** where microcephaly is reported in approximately 44% of patients [9], 2 out of 7 patients with duplication have macrocephaly. Such mirror phenotypes are often reported in the cases of microdeletion and microduplication syndromes of common region [15, 16].

The smallest region of overlap among reported cases encompasses 9 genes: *BCL11A, PAPOLG, REL, PUS10, PEX13,KIAA1841, C2orf74, ASHA2, USP34,* among them *BCL11A and PEX13* are OMIM Morbid genes. Gene PEX13 (OMIM*601789) is linked to the autosomal recessive Peroxisome biogenesis disorder 11A and 11B.

The second OMIM Morbid *BCL11A* has just recently been linked to new autosomal dominant Dias-Logan syndrome [17], with suggested clinical syndrome of at least mild dysmorphisms with intellectual disability and persistence of fetal haemoglobin. The type of mutation (missense mutations, truncating mutations, bigger deletions) partially defines the Dias-Logan phenotype, whereas the deletions of the neighbouring regions present as a 2p16.1p15 microdeletion syndrome with additional clinical features, as described above. The BCL11A is highly expressed in human cerebral cortex, hippocampus and cerebellum and the zebrafish knockdown models developed microcephaly and showed size reduction [18]. The 2p16.1p15 microdeletion syndrome patients have broad size range of deleted regions, and in addition to BCL11A, one more gene has shown potential link to the phenotype after extensive studies. Knockdown of REL in zebrafish resulted in specific structural brain anomalies, abnormal growth, and dysmorphisms. These findings are in accordance with known roles of REL in NF-kappaB pathway and memory formation [18].

When trying to decipher the meaning of duplications, search for copy number gain of selected genes was performed. There are currently no additional data (apart from the CNV sizes and some phenotype features of reported cases) available for the above mentioned genes in the literature. Telomeric borders of herein detailed duplications, including those from available databases with no specific phenotype data, do not differ by gene content (Fig. 2). On the centromeric border, there are 4 genes that are not uniformly duplicated in all cases, namely XPO1 (OMIM#602559), FAM16A1 (OMIM#613596), CCT4 (OMIM#605142) and COMMD1 (OMIM#607238). FAM16A1 is a known morbid gene, linked to autosomal recessive Retinitis pigmentosa 28. The other 3 genes have not been linked to human disease before and there are no individual phenotype features that could be linked to specific duplication sizes in the group of patients that are included in this report. Most likely explanation is that these genes do not cause significant additional clinical phenotype when present in 3 copies. Less likely, these might be increased gene dosage sensitive genes, but specific phenotype has not been sufficiently described in presented patients.

## Conclusion

Despite of almost two decades of molecular karyotyping in the group of individuals with developmental delay, intellectual disability, dysmorphic features and additional clinical characteristics, novel rare copy number variations are still identified. Their absence in the databases of normal copy number variation covering numerous populations renders further proof of potential significance or causality for specific phenotypes. Herein we report in detail 4 additional cases of 2p16.1p15 microduplication, which has been reported only once in the literature so far [11]. Each novel case of such a rare CNV provides important insights for clinicians, as well as for deciphering the human genome.

Together with previously reported data, our results suggest that the 2p16.1p15 microduplication might be linked to developmental delay, speech delay, mild to moderate intellectual disability, and unspecific dysmorphic features. Further cases are needed to decipher its clinical implications and decide if it represents a new clinical entity.
